# Influence of Match Location and Match Outcome on External Load Demands in Female Futsal Players

**DOI:** 10.3390/s26165241

**Published:** 2026-08-19

**Authors:** Rafael Albalad-Aiguabella, Oscar Villanueva-Guerrero, Alejandra Gutiérrez-Logroño, Alberto Roso-Moliner, Elena Mainer-Pardos

**Affiliations:** Health Sciences Faculty, University of San Jorge, Autov A23 km 299, 50830 Villanueva de Gállego, Zaragoza, Spain; ralbalad@usj.es (R.A.-A.); ovillanueva@usj.es (O.V.-G.); alejandraalg8@gmail.com (A.G.-L.); aroso@usj.es (A.R.-M.)

**Keywords:** female futsal, inertial measurement units, wearable sensors, accelerations, decelerations, contextual variables, performance, neuromuscular demands

## Abstract

Contextual factors may influence external load demands during futsal competition, yet evidence in women’s futsal remains limited. This study aimed to analyse the influence of match location and match outcome, as well as their interaction, on external load variables in female futsal players. Fourteen national-level female outfield players from a Spanish Second Division team were monitored across 27 official matches during the 2024–2025 season. External load variables were recorded using wearable inertial measurement units and normalised per minute of effective playing time. A total of 191 valid player-match observations were analysed using linear mixed models, with player identity and match identity included as crossed random intercepts. Neither match location nor match outcome significantly influenced any of the external load variables analysed, and no significant match location × match outcome interactions were observed (all *p* > 0.05). Nevertheless, descriptive differences and effect sizes ranging from trivial to large were observed across the analysed variables. These findings indicate that external load demands in female futsal remain relatively stable across match locations and final match outcomes. Future research including larger samples and more detailed situational variables is required to further investigate the potential influence of contextual factors on external load demands in women’s futsal.

## 1. Introduction

Futsal is a five-a-side indoor form of football played on a 40 × 20 m hard court, consisting of two 20-min halves with a stopped clock and unlimited substitutions, which contribute to the highly dynamic and high-intensity nature of the game [1]. The sport is characterized by repeated high-intensity actions performed in a limited space, making it one of the most physically demanding team sports. During a match, elite female futsal players repeatedly perform accelerations, decelerations, changes of direction (COD), and short maximal sprints, reflecting the intermittent high-intensity nature of the sport [2]. Despite the growing use of tracking technologies in team sports, studies examining external load demands in futsal remain relatively scarce, particularly in women’s competitions, highlighting the need for further research to better understand the external load demands of this population. Unlike football, futsal places greater emphasis on neuromuscular and anaerobic demands due to the frequency of high-intensity actions and the limited opportunities for prolonged recovery periods [3,4]. Players are required to complete 60–100 high-intensity efforts over 40 min of effective playing time while maintaining repeated sprint ability throughout the match. In addition, the small playing area, continuous involvement with the ball, rapid offensive–defensive transitions, and frequent scoring situations create distinctive mechanical and metabolic demands compared with traditional football [5,6,7].

Advances in athlete-monitoring technology have improved the analysis of external load in futsal. Because Global Positioning Systems (GPS) have limited accuracy in indoor environments, recent studies have adopted Local Positioning Systems (LPS), Ultra-Wideband Technology (UWB), and Inertial Measurement Units (IMUs) to monitor player movement and workload. In this context, IMUs have emerged as a practical solution for monitoring external load in indoor sports such as futsal, as they have demonstrated acceptable levels of validity and reliability for monitoring movement-related variables in intermittent team sports, such as accelerations, decelerations, and metabolic power [8,9]. These systems operate at high sampling frequencies and allow the accurate detection of short accelerations, decelerations, and rapid directional changes, which are characteristic of futsal. The use of these technologies has also facilitated the analysis of positional differences. For example, studies conducted in male futsal have reported that pivots tend to accumulate greater high-intensity running distances, whereas wingers perform a higher number of accelerations due to wider court coverage demands [10].

External load in futsal is commonly evaluated through locomotor, metabolic, and neuromuscular variables normalized to effective playing time (m·min^−1^ or n·min^−1^) [10]. Locomotor measures include total distance covered, high-intensity running distance, and sprint distance, which are associated with aerobic fitness and repeated sprint ability [11,12]. In addition, metabolic power has been proposed as a useful indicator for estimating the energetic demands of accelerations and CODs, which are not fully represented by speed thresholds alone [13]. Neuromuscular variables, particularly accelerations and decelerations above 2.5–3 m·s^−2^, are considered especially relevant because they produce high mechanical stress and have been linked to fatigue during competition [14,15].

Maximum speed and composite indicators such as equivalent distance and session load are also used to estimate overall match demands [16]. These variables generally demonstrate acceptable reliability across matches and help identify positional differences in external load demands [16]. For example, defenders tend to cover lower total distances but experience greater relative acceleration loads compared with other playing positions [7].

Contextual factors such as match location and match outcome could influence players’ external load responses during competition [17]. Research in futsal has shown that teams losing by multiple goals often increase high-intensity actions and accelerations in an attempt to regain possession and create scoring opportunities [18,19]. Similarly, players competing at home may exhibit higher work rates and sprint distances, potentially due to the absence of travel fatigue, crowd support, and familiarity with the playing environment [20]. However, the extent to which these contextual factors influence external load demands may vary depending on the specific characteristics of the sport [21].

The magnitude of these effects may vary depending on competition level, score progression, and tactical context [22,23]. Some studies indicate that external load intensity peaks during periods in which teams are attempting to overcome a deficit, whereas home teams experiencing losing scenarios may demonstrate mixed responses influenced by both tactical urgency and psychological factors [24,25]. In futsal, however, the influence of contextual variables may differ because indoor playing conditions are more standardized and the smaller court dimensions may influence tactical behavior and, consequently, external load demands [20].

Although external load has been extensively investigated in football, research in futsal remains limited, particularly in women’s competitions. Most available studies have focused on male players and have primarily used GPS- or LPS-based monitoring systems, despite the recent advantages of IMUs for monitoring external load in indoor sports environments. Existing findings regarding the effects of match outcome on external load demands are inconsistent, with some studies reporting greater physical demands in losing situations and others finding minimal differences [26]. Likewise, the influence of match location appears to be smaller in futsal than in football, possibly due to the controlled indoor environment [27]. Furthermore, limited evidence is available regarding how these contextual variables interact to influence external load demands in female futsal players.

Research on women’s futsal is especially scarce, despite evidence of sex-related differences in speed thresholds, acceleration profiles, and fatigue responses [7]. Furthermore, little information is available regarding the Spanish Second Division, which represents an important developmental level between amateur and elite competition. To date, no study has examined the interaction between match location and match outcome using linear mixed-model approaches applied to IMU-derived external load variables in female futsal players, highlighting an important gap in the literature.

Based on previous findings, it was hypothesized that matches resulting in losses would be associated with greater external load demands, particularly in variables related to high-intensity efforts, accelerations, and decelerations, due to the increased physical requirements of attempting to recover unfavorable scorelines. Furthermore, it was expected that players competing at home would exhibit greater external load values than those competing away, potentially as a consequence of the absence of travel fatigue, crowd support, and familiarity with the playing environment. Finally, given the limited evidence available in futsal, it was hypothesized that match location and match outcome would interact to influence external load responses in female futsal players. Therefore, the aim of this study was to analyze the influence of contextual variables (match location and match outcome), as well as their interaction, on external load variables in female futsal players from the Spanish Second Division. This study aims to provide novel insights into how contextual factors influence external load demands in female futsal.

## 2. Materials and Methods

### 2.1. Study Design

A longitudinal observational study design was employed to examine the physical performance of female futsal players, as assessed through external load demands, across an entire competitive season. Data were collected during the 2024–2025 season, from October 2024 to May 2025, including a total of 27 consecutive official matches (matchdays 4 to 30) from the Spanish Women’s Second Division. Data collection began from matchday 4 onwards because the first three league matches were considered part of the initial competitive adaptation period following the preseason. During this phase, players may still be adjusting to competitive demands, tactical roles, and match fitness levels, potentially resulting in atypical external load responses compared with the remainder of the season. Therefore, these matches were excluded to ensure that the analyzed data reflected more stable and representative competitive performance. Matches were played on a weekly basis, with data collection spanning from 13 October 2024 to 24 May 2025.

Throughout the observation period, both absolute and relative external load variables were recorded for all participating players. These variables included measures of locomotor activity (e.g., total distance and high-intensity running), neuromuscular demands (e.g., accelerations and decelerations), and composite load indicators (e.g., session load and intensity). Match outcome was distributed as 12 losses, 9 wins, and 6 draws, with the team finishing in 8th position out of 16 teams, corresponding to mid-table in the final league standings.

External load variables were continuously monitored during official competition using wearable inertial measurement units (IMUs), allowing for the quantification of players’ movement demands under real match conditions. Match location (home vs. away) and match outcome (win, draw, loss) were considered as independent variables, while external load variables were treated as dependent variables. The observational nature of the design ensured that no interventions were applied, preserving the ecological validity of the data collected in a high-performance competitive environment.

### 2.2. Participants

Fourteen highly trained national-level (TIER 3) [28] female futsal outfield players (age: 24.0 ± 5.65 years; height: 1.61 ± 0.05 m; body mass: 54.04 ± 4.87 kg; body mass index (BMI): 20.94 ± 1.84 kg/m^2^) were included in this study, from a team competing in the Spanish Women’s Second Division. The mean effective playing time per player per match was 17.29 ± 2.32 min.

To be included in the study, participants had to meet the following inclusion criteria: (i) be part of the club’s first team squad during the competitive season; (ii) participate as outfield players; and (iii) be available for official match participation without injury-related restrictions. Additionally, match observations were included only if the following criteria were met: (i) the player completed the match without withdrawal due to injury; (ii) the player accumulated at least 35% of the effective playing time; and (iii) the IMU data were complete and valid.

All participants were informed about the purpose and procedures of the study, and participation was voluntary. Written informed consent was obtained from all participants prior to data collection. For players under 18 years of age, informed consent was provided by their parents or legal guardians. The study was conducted in accordance with the Declaration of Helsinki and approved by the Ethics Committee of Universidad San Jorge (approval number: 126/1/24–25).

### 2.3. Instrumentation

External load variables were monitored using an OLIVER^TM^ inertial device (Oliver Sport, Barcelona, Spain). This system integrates an inertial measurement unit (IMU), including a triaxial accelerometer and gyroscope, together with a global navigation satellite system (GNSS), enabling the collection of movement-related data during match play.

The device records inertial data at 50 Hz and GNSS data at 25 Hz (Recorder and Analyzer, Barcelona, Spain), providing sufficient temporal resolution to capture the high-intensity and intermittent actions typical of futsal. Previous studies have reported acceptable validity and reliability of this technology for monitoring external load in futsal [9].

All matches analyzed in this study were performed in indoor sports facilities. Although GNSS signal reception may be reduced in indoor conditions, the combination of inertial sensors allows accurate monitoring of key external load variables such as accelerations, decelerations, and changes of direction, which are primarily derived from accelerometer data and therefore minimally affected by satellite signal limitations [8]. Furthermore, the OLIVER system has previously demonstrated acceptable validity for monitoring external load variables in futsal-specific indoor environments [9]. The device integrates inertial sensor data with proprietary processing algorithms to derive movement-related variables during match play, including distance-related metrics. However, the exact calculation procedures used by the manufacturer are not publicly available.

The device is lightweight (approximately 15 g) and compact, allowing players to wear it without affecting performance. It was secured in a custom-designed vest positioned in the interscapular region (upper back), ensuring stability and consistency of data acquisition during match play. Device configuration and data extraction were carried out using the manufacturer’s proprietary software (TryOliver Platform, version 2.54) [9].

### 2.4. Procedures

Players were monitored during official matches using the OLIVER^TM^ inertial device, following a standardized protocol to ensure consistency across all observations. Each player was assigned the same device throughout the season to minimize inter-unit variability.

The device was positioned as previously described. Prior to each match, devices were activated immediately after the warm-up, approximately 8–10 min before the start of the match, to ensure appropriate system initialization and signal acquisition.

External load variables were continuously recorded throughout match play. At the end of each match, the devices were switched off and removed by the players, and the recorded data were subsequently downloaded and processed using the manufacturer’s proprietary software (TryOliver Platform).

To ensure data quality, only recordings with complete data acquisition were included in the analysis. All procedures were conducted under consistent conditions across matches to minimize potential sources of measurement error.

Building on previous research conducted in futsal that has examined the physical and conditional demands of competition [1,9,10,29], the external load variables analyzed in this study were selected to provide a comprehensive representation of players’ locomotor, neuromuscular, and overall load demands.

Locomotor variables included maximum speed (km/h), total distance covered per minute (m·min^−1^), high-intensity running distance (m·min^−1^), and high-intensity running efforts (n·min^−1^). Neuromuscular demands were assessed through high-intensity acceleration distance (m·min^−1^), high-intensity accelerations (n·min^−1^), high-intensity deceleration distance (m·min^−1^), and high-intensity decelerations (n·min^−1^).

Additionally, composite load indicators were considered, including session load (arbitrary units: AU·min^−1^), defined as the total external load accumulated during match play based on the device’s internal algorithm, which integrates acceleration-derived data to quantify overall mechanical demands; session intensity (AU·min^−1^), calculated as a relative index reflecting the relationship between total load and match duration and representing the average external load per unit of time; and speed-based session intensity (AU·min^−1^) and acceleration-based session intensity (AU·min^−1^), which provide specific insights into the contribution of velocity- and acceleration-derived components to overall match intensity. As these composite load indicators are derived from proprietary algorithms implemented within the manufacturer’s software, their exact calculation formulas are not publicly available. Consequently, the reproducibility and direct comparison of these metrics across different tracking systems may be limited. All external load variables, except maximum speed, were normalized per minute of effective playing time to account for differences in individual exposure and the intermittent nature of futsal competition.

### 2.5. Statistical Analysis

All statistical analyses were performed using SPSS (IBM SPSS Statistics, Version 29, IBM Corp., Armonk, NY, USA). A total of 247 match observations were initially collected. After applying an inclusion criterion requiring participation in at least 35% of the effective playing time, 191 observations were retained for analysis, following previous methodological approaches in futsal research [15]. All observations were obtained from official competitive matches and represented individual player match performances. Descriptive statistics are presented as mean ± standard deviation (SD). Normality of the data distribution was examined using the Shapiro–Wilk test. The assumptions of the linear mixed models were subsequently evaluated by visual inspection of residual plots and normal Q–Q plots to assess residual normality and homoscedasticity. No substantial violations of these assumptions were identified. Due to the repeated-measures design and the crossed structure of the data, linear mixed models were applied, as this approach is suitable for accounting for within-subject correlations and handling unbalanced datasets [30]. To appropriately account for the hierarchical structure of the data and avoid pseudoreplication, a crossed random-effects structure was specified. External load variables were treated as dependent variables, while match location (home vs. away) and match outcome (win, draw, loss) were included as fixed factors. Player identity and match identity were included as crossed random intercepts to account for repeated observations within players and the clustering of observations within matches. A random-intercept structure was selected because the primary objective was to account for the correlation of repeated observations while estimating the average fixed effects of match location and match outcome. More complex random-effects structures (e.g., random slopes) were not adopted because the relatively small number of players and matches could have resulted in model overparameterisation and unstable parameter estimates. Models were estimated using restricted maximum likelihood (REML). Fixed effects were evaluated using Type III F-tests, and statistical significance was set at *p* < 0.05. Estimated marginal means and their corresponding 95% confidence intervals were obtained for the fixed effects of match location and match outcome. No formal adjustment for multiple comparisons was applied because the analysed external load variables represent related but distinct performance outcomes. Consequently, the findings were interpreted considering the overall pattern of results rather than isolated statistical significance. Effect sizes for the fixed effects were calculated using partial eta squared (*ηp*^2^), derived from the Type III F-tests of the linear mixed models, and interpreted according to conventional thresholds as trivial (<0.01), small (0.01–0.05), moderate (0.06–0.13), and large (≥0.14) [31].

## 3. Results

Descriptive statistics for all external load variables are presented in Table 1.

Overall, players exhibited moderate-to-high external load demands during competition, with total distance averaging 55.18 ± 11.13 m·min^−1^ and a mean maximum speed of 20.25 ± 2.07 km·h^−1^. Neuromuscular variables, including accelerations and decelerations, also showed considerable variability across match observations, reflecting the intermittent and high-intensity nature of futsal.

The effects of match location, match outcome, and their interaction on external load variables are presented in Table 2, whereas the estimated marginal means, together with their corresponding 95% confidence intervals derived from the linear mixed models, are presented in Table 3.

No significant main effects of match location or match outcome were observed for any of the variables analyzed (all *p* > 0.05). Likewise, no significant interaction effects between match location and match outcome were found (all *p* > 0.05). Effect sizes ranged from trivial to large. The largest effect sizes were observed for the match location × match outcome interaction in maximum speed (*ηp*^2^ = 0.200), the main effect of match outcome on total distance (*ηp*^2^ = 0.186) and session load (*ηp*^2^ = 0.160), and the match location × match outcome interaction in high-intensity decelerations (*ηp*^2^ = 0.160). Nevertheless, none of these effects reached statistical significance.

## 4. Discussion

The aim of this study was to analyze the influence of contextual variables (match location and match outcome), as well as their interaction, on external load variables in female futsal players. Overall, the results showed that neither match location nor match outcome significantly influenced any of the external load variables analyzed. Similarly, no significant interaction effects between match location and match outcome were observed for any of the variables. In contrast, descriptive differences were observed across some variables according to match outcome and match location; however, these differences did not reach statistical significance in the linear mixed models. The effect sizes associated with match location, match outcome, and their interaction ranged from trivial to large. However, the larger effect-size estimates were not accompanied by statistically significant fixed effects and should therefore be interpreted cautiously. Consequently, the present findings do not provide statistical evidence supporting an influence of these contextual factors on the external load variables analysed. Overall, these findings suggest that no statistically significant differences in external load demands were observed across the contextual conditions examined within the analysed sample. This pattern is consistent with previous futsal research showing that contextual factors may have a limited effect on external load when the demands of the game are strongly constrained by the indoor nature of futsal and the reduced playing area [15,20].

The non-significant effects observed for match outcome suggest that the final score did not meaningfully influence players’ external load responses during the match. Although descriptive differences were observed between winning, drawing, and losing situations, these variations did not reach statistical significance in the linear mixed models. This indicates that, within the analyzed sample, external load demands remained relatively stable regardless of match status. Previous studies conducted with male futsal players have similarly reported that external load demands were relatively stable irrespective of match status [15,20]. Therefore, from a theoretical perspective, it could be expected that teams facing a disadvantage would increase their physical effort in an attempt to reverse the result; however, the present findings do not statistically support this assumption. Although moderate-to-large effect-size estimates were observed for some variables, these findings should be interpreted cautiously in light of the relatively small sample size and the inherent variability of competitive match performance. This possibility has been supported by some studies, which have shown that match status can influence players’ performance, particularly when teams are under pressure to overturn the scoreline or respond to an unfavorable result [25,32]. Furthermore, studies by Ribeiro et al. and Spyrou et al. reported that futsal is characterized by rapid transitions and actions constrained by space, time, and tactical adjustments, making the influence of match outcome on overall external load smaller than that observed in other sports [7,26].

Although no significant effects of match outcome were identified, neuromuscular variables remain particularly relevant for characterising external load in this sport. In futsal, performance is characterized by repeated accelerations, decelerations, and changes of direction within a confined playing area, making neuromuscular actions particularly relevant for load characterization [10]. In this regard, variables such as high-intensity accelerations and decelerations may be more sensitive to contextual fluctuations than total distance covered [20]. However, in the present study, these variables did not differ significantly according to match outcome. Therefore, although neuromuscular variables remain important descriptors of external load in futsal, the present findings do not provide sufficient statistical evidence to conclude that they are meaningfully influenced by final match outcome.

Similarly, the absence of significant effects of match location may be explained by the specific nature of futsal. Given that court dimensions, playing surface, and match environment are essentially the same in both home and away matches, the influence of match location is likely to be smaller than that observed in outdoor football [20]. Furthermore, the indoor setting, the relatively smaller number of spectators, and the highly structured tactical dynamics of the game may reduce the impact of crowd pressure and the traditional mechanisms associated with home advantage [33]. This explanation is supported by previous futsal studies reporting that contextual factors such as match location do not always modify external load demands, even when considered alongside match outcome and team ranking [15,20]. However, some league-level futsal studies have identified a home advantage in terms of match outcomes, suggesting that match location may influence competitive performance without necessarily affecting external load responses [33]. Therefore, the absence of location effects in the present study should not be interpreted as evidence that home advantage does not exist. Rather, it may indicate that its influence is weaker or less relevant for external load variables within the specific context of women’s futsal.

Overall, the present findings are partially consistent with previous research in futsal and football [34,35]. Although studies conducted in football have reported more pronounced effects of contextual variables, particularly match status and match location, these influences may be attenuated in futsal because of the sport’s more constrained and highly standardised competitive environment [36,37]. The absence of statistically significant effects indicates that external load responses were relatively stable across contextual conditions in this sample of female futsal players. Nevertheless, contextual factors not considered in the present study, such as opposition quality, score progression throughout the match, and substitution patterns, may also contribute to external load variability and should be explored in future research. In this regard, futsal may be more sensitive to immediate tactical and situational factors than to broader contextual variables such as match location or final match outcome, particularly with respect to quantifiable external load demands.

Several limitations should be considered when interpreting these findings. First, the sample was based on a single team, which limits the generalizability of the results to other competitive levels, playing styles, or leagues. Furthermore, although repeated player–match observations increased the amount of information available for within-team analyses, the relatively small number of players may have reduced the statistical power to detect subtle contextual effects. Second, only external load variables were analyzed; therefore, future studies should incorporate internal load measures, tactical behavior, and psychological factors to provide a more comprehensive understanding of the mechanisms underlying players’ responses to contextual variables. Third, the study did not directly assess contextual factors such as score progression during the match, opposition quality, or substitution patterns, all of which may influence external load responses in futsal and help explain potential contextual variations. In addition, some composite load indicators (e.g., session load, session intensity, speed-based session intensity, and acceleration-based session intensity) were derived from proprietary algorithms provided by the manufacturer. As the exact calculation procedures are not publicly available, the reproducibility and direct comparison of these variables across different monitoring systems may be limited. Therefore, the present findings should be interpreted within the methodological constraints of the study, particularly regarding statistical power and external validity.

From a practical perspective, the present findings suggest that external load demands in women’s futsal remain relatively stable across different match locations and match outcomes. Therefore, coaches may prioritize training strategies based on the overall physical and tactical demands of the game rather than adjusting external load expectations according to match location or final match outcome. Although the present findings do not support a meaningful influence of these contextual variables on external load, designing training tasks that include repeated accelerations, decelerations, changes of direction, and high-intensity actions may help players cope with the variability inherent to competitive match play. These findings provide valuable applied information for practitioners working in women’s futsal.

## 5. Conclusions

This study showed that neither match outcome nor match location significantly influenced the external load demands experienced by female futsal players. Similarly, no significant interaction effects between match outcome and match location were observed for any of the analyzed variables. Overall, the findings suggest that no statistically significant differences in external load responses were observed across the contextual conditions examined within the analysed sample.

These findings do not provide sufficient statistical evidence to conclude that contextual factors meaningfully influenced external load demands within the analysed sample. From an applied perspective, coaches and performance staff may prioritise training strategies based on the overall physical and tactical demands of the game rather than on match location or final match outcome. Nevertheless, training programmes should continue to emphasise repeated accelerations, decelerations, changes of direction, and other high-intensity actions, as these represent key physical demands of futsal performance. Future research should include larger samples, multiple teams, and additional contextual variables such as score progression, opposition quality, tactical behaviour, and internal load responses to better understand the factors influencing external load fluctuations in women’s futsal competition.

## Figures and Tables

**Table 1:** Descriptive statistics of all external load variables (mean ± SD).

Variable	Min	Max	Mean	SD
Maximum speed (km·h^−1^)	11.00	24.40	20.25	2.07
Total distance (m·min^−1^)	31.49	92.13	55.18	11.13
High-intensity running distance (m·min^−1^)	0.33	9.11	3.02	1.66
High-intensity running efforts (n·min^−1^)	0.08	1.44	0.46	0.23
High-intensity acceleration distance (m·min^−1^)	0.80	8.70	2.88	1.27
High-intensity accelerations (n·min^−1^)	0.11	1.06	0.37	0.15
High-intensity deceleration distance (m·min^−1^)	1.24	7.80	3.59	1.29
High-intensity decelerations (n·min^−1^)	0.22	1.42	0.75	0.24
Session load (AU·min^−1^)	0.74	2.57	1.41	0.34
Session intensity (AU·min^−1^)	0.57	7.14	1.60	0.85
Speed-based session intensity (AU·min^−1^)	0.64	6.99	1.75	0.86
Acceleration-based session intensity (AU·min^−1^)	0.43	7.36	1.46	0.88

SD: Standard Deviation.

**Table 2:** Descriptive statistics (mean ± SD), fixed effects, and effect sizes (*ηp*^2^) from the linear mixed models according to match location and match outcome.

Variable	HOME	AWAY	WIN	DRAW	LOSS	Location (F, *p*)	Location *ηp*^2^	Outcome (F, *p*)	Outcome *ηp*^2^	Location × Outcome (F, *p*)	Location × Outcome *ηp*^2^
Maximum speed (km·h^−1^)	20.41 ± 2.27	20.10 ± 1.87	20.19 ± 1.98	20.25 ± 2.41	20.30 ± 2.00	2.026, 0.169	0.086	1.224, 0.314	0.102	2.706, 0.089	0.2
Total distance (m·min^−1^)	53.84 ± 10.71	56.41 ± 11.42	52.82 ± 10.53	54.74 ± 11.68	57.36 ± 11.06	1.111, 0.304	0.049	2.435, 0.112	0.186	1.506, 0.244	0.123
High-intensity running distance (m·min^−1^)	3.00 ± 1.62	3.03 ± 1.70	2.92 ± 1.65	3.05 ± 1.60	3.09 ± 1.71	0.112, 0.741	0.006	0.512, 0.607	0.051	1.678, 0.213	0.147
High-intensity running efforts (n·min^−1^)	0.46 ± 0.24	0.46 ± 0.23	0.46 ± 0.25	0.45 ± 0.21	0.48 ± 0.24	0.152, 0.701	0.008	0.363, 0.700	0.036	1.001, 0.385	0.091
High-intensity acceleration distance (m·min^−1^)	2.85 ± 1.28	2.91 ± 1.27	2.77 ± 1.15	2.67 ± 1.11	3.08 ± 1.42	0.719, 0.407	0.035	0.652, 0.532	0.062	0.699, 0.509	0.067
High-intensity accelerations (n·min^−1^)	0.36 ± 0.15	0.37 ± 0.15	0.35 ± 0.14	0.34 ± 0.13	0.39 ± 0.16	0.388, 0.541	0.019	0.687, 0.515	0.066	0.362, 0.701	0.035
High-intensity deceleration distance (m·min^−1^)	3.62 ± 1.32	3.56 ± 1.26	3.43 ± 1.29	3.74 ± 1.27	3.66 ± 1.29	0.310, 0.584	0.014	1.163, 0.331	0.097	1.252, 0.306	0.105
High-intensity decelerations (n·min^−1^)	0.75 ± 0.24	0.75 ± 0.24	0.71 ± 0.24	0.78 ± 0.24	0.76 ± 0.24	0.107, 0.746	0.006	1.259, 0.305	0.06	0.906, 0.420	0.16
Session load (AU·min^−1^)	1.38 ± 0.35	1.43 ± 0.34	1.35 ± 0.34	1.45 ± 0.36	1.43 ± 0.34	0.818, 0.375	0.036	2.131, 0.142	0.16	1.500, 0.245	0.12
Session intensity (AU·min^−1^)	1.54 ± 0.77	1.66 ± 0.91	1.59 ± 0.93	1.43 ± 0.58	1.70 ± 0.88	2.464, 0.131	0.105	0.543, 0.589	0.049	1.667, 0.213	0.137
Speed-based session intensity (AU·min^−1^)	1.66 ± 0.75	1.82 ± 0.94	1.71 ± 0.91	1.58 ± 0.62	1.86 ± 0.90	0.972, 0.326	0.053	1.265, 0.285	0.03	1.820, 0.165	0.06
Acceleration-based session intensity (AU·min^−1^)	1.42 ± 0.82	1.50 ± 0.93	1.48 ± 0.98	1.28 ± 0.55	1.54 ± 0.90	0.298, 0.586	0.013	1.026, 0.361	0.035	0.350, 0.705	0.038

Note: Values are presented as mean ± standard deviation. Fixed effects were obtained from linear mixed models including match location and match outcome as fixed factors and player identity and match identity as crossed random effects.

**Table 3:** Estimated marginal means (EMMs) and 95% confidence intervals (95% CI) from the linear mixed models according to match location and match outcome.

Variable	HOME Mean (95% CI)	AWAY Mean (95% CI)	WIN Mean (95% CI)	DRAW Mean (95% CI)	LOSS Mean (95% CI)
Maximum speed (km·h^−1^)	20.4 (19.5–21.3)	20.1 (19.4–20.8)	20.2 (19.4–21.0)	20.3 (19.3–21.2)	20.3 (19.5–21.1)
Total distance (m·min^−1^)	51.8 (47.1–56.5)	53.5 (48.8–58.2)	50.5 (45.7–55.4)	52.9 (47.8–58.1)	54.5 (49.8–59.3)
High-intensity running distance (m·min^−1^)	2.91 (2.20–3.61)	2.96 (2.25–3.67)	2.82 (2.10–3.54)	3.01 (2.27–3.75)	2.98 (2.26–3.69)
High-intensity running efforts (n·min^−1^)	0.45 (0.35–0.55)	0.46 (0.36–0.56)	0.44 (0.34–0.55)	0.45 (0.34–0.55)	0.46 (0.36–0.57)
High-intensity acceleration distance (m·min^−1^)	2.82 (2.26–3.39)	2.91 (2.34–3.47)	2.75 (2.16–3.34)	2.72 (2.10–3.35)	3.13 (2.56–3.70)
High-intensity accelerations (n·min^−1^)	0.36 (0.29–0.43)	0.37 (0.30–0.44)	0.35 (0.28–0.42)	0.35 (0.27–0.43)	0.40 (0.33–0.47)
High-intensity deceleration distance (m·min^−1^)	3.43 (2.95–3.90)	3.39 (2.92–3.87)	3.20 (2.70–3.70)	3.56 (3.02–4.11)	3.47 (2.98–3.95)
High-intensity decelerations (n·min^−1^)	0.72 (0.63–0.81)	0.72 (0.63–0.81)	0.68 (0.58–0.77)	0.75 (0.64–0.85)	0.73 (0.64–0.83)
Session load (AU·min^−1^)	1.34 (1.21–1.46)	1.37 (1.24–1.49)	1.28 (1.14–1.41)	1.42 (1.27–1.56)	1.36 (1.23–1.49)
Session intensity (AU·min^−1^)	1.77 (1.25–2.29)	1.88 (1.36–2.39)	1.87 (1.35–2.40)	1.75 (1.21–2.29)	1.84 (1.32–2.37)
Speed-based session intensity (AU·min^−1^)	1.75 (1.34–2.16)	1.90 (1.49–2.31)	1.85 (1.42–2.27)	1.75 (1.30–2.19)	1.88 (1.47–2.29)
Acceleration-based session intensity (AU·min^−1^)	1.78 (1.13–2.42)	1.85 (1.20–2.49)	1.89 (1.24–2.54)	1.74 (1.08–2.41)	1.80 (1.16–2.45)

## Data Availability

Data supporting the findings of this research are available from the corresponding author (epardos@usj.es) upon reasonable request.

## Abbreviations

The following abbreviations are used in this manuscript:

AU	Arbitrary units
COD	Change of direction
GNSS	Global Navigation Satellite System
GPS	Global Positioning System
IMU	Inertial Measurement Unit
LMM	Linear mixed model
LPS	Local Positioning System
REML	Restricted Maximum Likelihood
SD	Standard deviation
UWB	Ultra-wideband
